# Safeguarding nurses' mental health: The critical role of psychosocial safety climate in mitigating relational stressors and exhaustion

**DOI:** 10.3934/publichealth.2024046

**Published:** 2024-07-16

**Authors:** Teresa Galanti, Michela Cortini, Giuseppe Filippo Giudice, Salvatore Zappalà, Ferdinando Toscano

**Affiliations:** 1 Department of Psychology, University “Gabriele d'Annunzio” of Chieti-Pescara, Chieti (CH), Italy; 2 Department of Cardiovascular Diseases, “SS. Annunziata” Hospital, Chieti (CH), Italy; 3 Department of Psychology “Renzo Canestrari”, Alma Mater Studiorum University of Bologna, Cesena (FC), Italy; 4 Department of Psychology, University of Campania “Luigi Vanvitelli”, Caserta (CE), Italy

**Keywords:** psychological safety climate, relational stressors, exhaustion, burnout, nurses, mental health

## Abstract

Burnout among nurses is a pervasive concern in healthcare, with profound implications for patient care and nurse well-being. While research has highlighted the detrimental effects of burnout on many aspects of nursing, including patient safety and quality of care, the underlying mechanisms driving burnout warrant further investigation. In this cross-sectional study, we surveyed 196 nurses from diverse Italian hospitals using an online questionnaire via Qualtrics. Our findings revealed significant negative correlations between psychological safety climate and both relational stressors and emotional exhaustion. Conversely, relational stressors positively correlated with emotional exhaustion, and a significant negative indirect effect of psychological safety climate was found for emotional exhaustion through relational stressors, emphasizing the pivotal role of psychological safety climate in mitigating nurse burnout. Our study underscores the potential effectiveness of interventions targeting psychological safety climate and relational stressors in alleviating emotional exhaustion and burnout among nurses. Theoretical implications underscore the importance of deepening the role of psychological safety climate in mitigating emotional exhaustion, while practical implications emphasize the need for fostering a positive psychological safety climate and implementing targeted interventions to support nurses' well-being.

## 1. Introduction

In the complex world of their profession, nursing staff face a widespread challenge resulting from prolonged exposure to work-related stressors, namely burnout [1]–[3]. This syndrome exacts a toll across cognitive, emotional, and attitudinal domains, profoundly influencing behaviors towards work, colleagues, patients, and the very essence of the professional role [4]. Over time, scholars have put forth different conceptualizations of burnout. The traditional approach pioneered by Maslach and Jackson [5] has laid the groundwork for comprehending burnout highlighting emotional exhaustion, depersonalization, and reduced personal accomplishment as its fundamental components [6]. While emotional exhaustion involves feeling drained emotionally, cynicism entails developing a sense of detachment towards work, colleagues, and patients. On the other hand, reduced personal accomplishment refers to experiencing a diminished sense of achievement and competence in both professional and personal domains [6].

Subsequent research on burnout has led to refinements, such as those proposed by Salanova and colleagues, who emphasized exhaustion and suggested combining cynicism and depersonalization into a single construct called “mental distance” [7]. Despite the multi-componential nature of burnout, and its specific classifications, research has consistently placed significant emphasis on exhaustion, due to its debilitating effects on cognitive processes, emotional regulation, and overall energy levels, as well as for its power to predict the other burnout dimensions [8],[9].

Prior studies have revealed that exhaustion stems from work-related factors such as work overload, lack of social support, and inadequate rewards [2],[4], and in turn significantly impacts critical outcomes including patient safety, quality of care, nurses' organizational commitment, productivity, and patient satisfaction [10]. Given its wide-ranging implications, investigating deeper nurse exhaustion is paramount, and identifying its potential triggers is crucial for mitigating the risk of burnout occurrence.

Alongside the demands of their work rhythms and the conditions that arise within healthcare facilities, the nature of interaction and engagement with others significantly contributes to the emergence of exhaustion among nurses. The relational aspect of nursing, characterized by constant interaction with patients, families, and colleagues, places emotional and psychological demands on these professionals. During their work, nurses not only experience positive interactions leading to positive experiences, such as for instance the experience of receiving gratitude [11],[12], but also face the risk of verbal, physical, or emotional aggression in their workplace environments [13]. Research has shown that healthcare settings can be high-stress environments where tensions can escalate, leading to instances of aggression directed towards healthcare professionals [14]. Many employees in healthcare mistakenly perceive workplace violence as an inevitable aspect of their work environment [14],[15]. Furthermore, there is a prevalent belief among staff members that perpetrators of violence are unlikely to face any repercussions for their actions [16], making the importance of the work environment critical in determining the onset of burnout risk.

Individual perceptions of organizational factors can then play a role in exacerbating the risk of burnout in nurses. Inadequate staffing levels, long working hours, and inappropriate security measures have been extensively cited as burnout development facilitators by previous research [17]–[19]. Despite this, the relationship between individual perceptions of organizational factors and relational stressors in the context of nursing burnout warrants closer examination, as it remains an underexplored aspect within existing research.

Specifically, hospitals that fail to prioritize the security and safety of their nursing staff risk exacerbating interpersonal tensions, fostering suboptimal communication channels, and undermining support structures within the workplace. This, in turn, can escalate conflicts with patients, as well as their families and friends, leading to significant mental health strain on nurses.

Psychological safety climate (PSC) encapsulates the individual perceptions on the collective assumptions within a workplace regarding policies, practices, and procedures concerning employees' psychological health and safety [20]. Based on this definition and on a vision of climate with an individual level referent [21], we claim that psychological safety climate represents a unique reflection on the individual of the organizational atmosphere, shaped by individuals' perceptions of safety within the workplace. It serves as a compass for organizational conduct, impacting group decisions regarding the implementation of safety protocols, adherence to regulations, and compliance with instructions related to equipment usage. These individual perceptions are primarily shaped by the management and lived group dynamics, making PSC an upstream resource crucial for preventing and managing psychosocial workplace injuries. PSC comprises four key subdimensions, which reflects (a) management's commitment to psychological health, (b) prioritization of psychological well-being alongside productivity goals, (c) effective communication, including receptivity to employee concerns, and (d) active participation and consultation of all stakeholders in mitigating psychosocial risks and enhancing well-being. Previous studies have emphasized the interconnectedness between perceived safety and health climates and several factors, such as safety practices, motivation, knowledge, accidents, and overall physical health and well-being [22]. Moreover, an emerging body of research underscores the significance of a psychological safety climate in fostering favorable health and well-being outcomes. As highlighted by Clarke [23], an unfavorable perception of the psychological safety climate correlates with heightened stress levels and diminish psychological well-being.

Inadequate security measures contribute to an environment where nurses feel vulnerable, impeding their ability to effectively engage with patients and their support networks. Consequently, strained interactions and heightened stress levels among nurses can precipitate a cycle of relational tension, eroding the quality of patient care and amplifying the risk of burnout.

While previous evidence has already shown a negative relationship between psychological safety climate and burnout dimensions in health-care settings, with a relevant focus on exhaustion [24],[25], the underlying mechanisms driving this association remain relatively unexplored. One potential mechanism underlying this relationship lies within patient-related social stressors [26], identified by literature as among the most significant factors affecting nurses' well-being [27]. Psychological safety climate encompasses the psychological strain experienced by individuals due to interpersonal interactions, social expectations, and perceived social threats within healthcare settings. This stress can arise from various sources, such as social conflicts, injustice, unfair treatment or nonreciprocal behavior, and antisocial behavior at work [28].

Given the multifaceted nature of the social stressors discussed, it is crucial to consider how healthcare institutions can support nurses in managing their interactions with patients and families. Adamis and colleagues [29] highlight the concept of “patient-related burnout” underscoring the significant impact of patient interactions on nurses' well-being. Similarly, Dormann and Zapf [26] suggest that burnout may arise when nurses struggle to effectively manage these interactions. Considering these insights, it becomes evident that creating a supportive environment within hospital settings is essential and to ascertain whether fostering an environment where nurses feel safe makes them equipped to manage challenging relational situations without succumbing to exhaustion. For this reason, in accordance with the cited research framework on psychological safety climate [20] and the Job Demands-Resources Model [30], in this paper we aim to evaluate the mediating role of nurses' relational stressors in the relationship between nurses' psychological safety climate and nurses' exhaustion. We therefore present the methodology and results of our study below, and then we discuss them thus suggesting theoretical and practical implications.

## 2. Materials and methods

### 2.1. Participants and procedure

This cross-sectional study involved a sample of 196 nurses working in Italian hospitals. Data were collected via an online questionnaire distributed through the Qualtrics platform. Initially, one of the researchers circulated the questionnaire within his professional network, inviting nurses to participate in the study. The dissemination of the questionnaire began with two key colleagues: one working in a hospital in central-southern Italy and another in a hospital in northern Italy. In turn, the participating nurses involved other colleagues by inviting them to participate through direct presentation of the study and social media contacts. To ensure participants' perception of anonymity, we did not record the specific hospitals where the respondents were employed.

Despite their self-selection, the number of study participants exceeded the sample size required for the research model, as determined by an a priori evaluation using G*Power software. This evaluation considered the type of analysis, the number of predictors, a medium effect size (*F²* = 0.15), an alpha error probability (*α*) of 0.05, and a power (1-*β*) of 0.95. The calculation indicated that a sample size of approximately 119 participants could have been sufficient.

Prior to completing the questionnaire, each participant was informed of the objectives of the study, the anonymous nature of the survey, and the right to drop participation in the research at any time without consequence. In addition, each participant gave his or her consent to participate in the study and to have his or her data managed for the purposes of the research.

Since no manipulations were carried out and no sensitive questions were asked, according to the national law, no ethical approval was required. However, all ethical guidelines on social sciences research and the Declaration of Helsinki were followed.

### 2.2. Measures

In this study, the following scales were used to collect answers.

Psychological safety climate was measured using the three-item scale of safety climate created by Neal and Griffin [31]. Items were measured on a 7-point rating scale ranging from 1 (strongly disagree) to 7 (strongly agree). An example of an item is “Management places a strong emphasis on workplace health and safety”. In this study, both Cronbach's alpha and McDonald's omega values were 0.96 for this scale.

Relational stressors was assessed through the 12 items of the scale by Dormann and Zapf [26]. Items were measured on a 7-point rating scale ranging from 1 (strongly disagree) to 7 (strongly agree). An example of an item is “Patients are always complaining about us”. In this study, both Cronbach's alpha and McDonald's omega values were 0.91 for this scale.

Emotional exhaustion was measured through the four items subscale of emotional exhaustion of the Spanish Burnout Inventory (SBI) by Gil-Monte and Figueiredo-Ferraz [32]. Items were measured on a 5-point rating scale ranging from 1 (never) to 5 (very frequently). An example of an item is “I feel I am overwhelmed by work”. In this study, both Cronbach's alpha and McDonald's omega values were 0.88 for this scale.

### 2.3. Data analysis

Before calculating the research model, we evaluated the effect of a Common Method Bias through the Harman test by employing exploratory factor analysis (EFA) and the principal axis method. Thus, we assessed the measurement model, the structural validity and reliability of our measures by running two confirmatory factor analyses (CFAs) and computing Cronbach's alpha, McDonald's omega (ω), and the Average Variance Extracted (AVE) values for the study scales. We then computed descriptive statistics (average and standard deviation) for evaluating the collected participants' personal characteristics and their perceptions, and correlations among variables. Finally, we tested our hypotheses using the Jamovi medmod module. All analyses were performed using Jamovi 2.3.

## 3. Results

### 3.1. Validity and reliability of the scales

A single-factor EFA was conducted as part of a Harman test to assess the potential presence of common method bias in our dataset. The results of the test indicated that the extracted single factor accounted for 33.38% of the variance, falling below the commonly accepted threshold of 50% utilized in previous studies [33]. This suggests that there may be no significant common method bias present in our data. To test the structural independence of the three measures of our model and the absence of a common latent factor, we conducted two CFAs, comparing a 1-factor model, in which all items were grouped, with a 3-factor model, with each item put under its expected factor.

The 1-factor model fit, as expected, was not good (chi-squared (*χ^2^*) = 1247.24; degrees of freedom (df) = 152; *χ^2^*/*df* = 8.20; Comparative Fit Index (CFI) = 0.47; Tucker-Lewis Index (TLI) = 0.41; Root Mean Square Error of Approximation (RMSEA) = 0.21; Standardized Root Mean Squared Residual (SRMR) = 0.15), while the 3-factor model was good (*χ^2^* = 394.19; *df* = 149; *χ^2^*/*df* = 2.65; CFI = 0.88; TLI = 0.86; RMSEA = 0.10; SRMR = 0.06). The model improved further when two pairs of items from the exhaustion factor were allowed to correlate (*χ^2^* = 291.65; *df* = 147; *χ^2^*/*df* = 1.98; CFI = 0.93; TLI = 0.92; RMSEA = 0.08; SRMR = 0.05), thus confirming the validity of our model. Additionally, the reliability (α and ω) and validity (AVE) values, reported in Table 1, showed no concerns in terms of psychometric properties of the adopted scales.

### 3.2. Description of participants and variables descriptive statistics

The study participants primarily consisted of women (57%) with an average age of 43.49 years [standard deviation (*SD*) = 11.95; ranging from 22 to 65]. On average, participants had been with their current organization for 14.78 years (*SD* = 11.99; ranging from 0 to 42). The majority did not reside with children under the age of 14 (76.5%), and only a small fraction (4.1%) had coordinating responsibilities on colleagues.

Descriptive statistics revealed moderate levels of psychological safety climate (*M* = 3.95; *SD* = 1.86) and relatively elevated levels of relational stressors (*M* = 4.93; *SD* = 1.20). Emotional exhaustion was also reported at moderate levels (*M* = 3.23; *SD* = 0.91). The correlation matrix showed significant correlations among all the study variables (see Table 1).

**Table 1.** Reliability Indices, Descriptive Statistics and Correlations among Variables.

**Variable**	**Alpha**	**Omega**	**AVE**	***M***	***SD***	**1**	**2**
1. Psychological safety climate	0.96	0.96	0.46	3.95	1.86		
2. Relational stressors	0.91	0.91	0.67	4.93	1.20	-0.22**	
3. Emotional exhaustion	0.88	0.88	0.88	3.23	0.91	-0.40**	0.35**

Note: **p < 0.01.

### 3.3. Model testing

**Figure 1.**
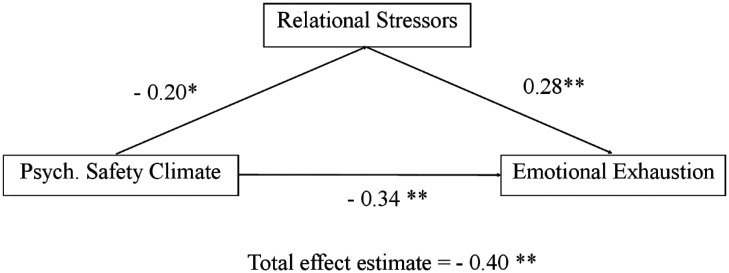
Standardized research model results.

In accordance with our research aims, we tested a mediation model in which psychological safety climate resulted significantly and negatively correlated with both relational stressors (*B* = -0.13; *SE* = 0.05; *β* = -0.20; *p* = 0.02) and emotional exhaustion (*B* = -0.17; *SE* = 0.04; *β* = -0.34; *p* < 0.001). On the other hand, the relationship between relational stressors and emotional exhaustion was positive (*B* = 0.22; *SE* = 0.06; *β* = 0.28; *p* < 0.001). Finally, the indirect effect of psychological safety climate on emotional exhaustion through relational stressors was also significant and negative (estimate = -0.03; *SE* = 0.01; standardized estimate = -0.06; *p* = 0.05), making the total effect of psychological safety climate on emotional exhaustion furtherly negative, and significant (estimate = -0.20; *SE* = 0.04; standardized estimate = 0.40; *p* < 0.001). In terms of explained variance calculated according to the guidelines by Fairchild and colleagues [34], the whole model explained approximately 16% of the variance in emotional exhaustion. The mediating effect alone explained approximately 7.84% of the variance in emotional exhaustion through the partial mediation of relational stressors. Figure 1 depicts the research model with results.

## 4. Discussion

We aimed to explore the relationships between psychological safety climate, relational stressors, and emotional exhaustion among Italian nurses, employing a mediation model. Our findings support the hypothesis that a favorable PSC, studied at an individual level perspective, is linked to reduced levels of perceived relational stressors and emotional exhaustion among nurses. Consistent with prior research [35]–[37], our study underscores the importance of perceived organizational support in equipping nurses to manage stressors effectively. Specifically, it highlights how a safety-oriented organizational climate can reduce the perception of social stressors, thereby mitigating the emotional strain experienced by nurses in their daily work. Moreover, this study prompts a critical discussion about the tangible benefits derived from investing in safety management for organizations. In fact, the costs associated with nurses' stress and burnout may include increased absenteeism [38] and reduced quality of care [39], resulting in higher rates of medical complications, prolonged hospital stays, and readmissions, all of which can incur additional costs for the organization. Reduced staff satisfaction due to stressful working conditions can negatively impact motivation and commitment [40]. Furthermore, staff turnover driven by stress and burnout necessitates further resources also for the hospital management boards, as for instance happen for recruitment, training, and onboarding of new nurses, potentially disrupting continuity and quality of care [41].

Our findings are consistent with earlier studies that examined the impact of PSC on stress and burnout. For example, Neal and Griffin [31] found that safety climate perceptions were negatively correlated with stress and positively correlated with job satisfaction. Similarly, Dollard and Bakker [42] proposed that a positive PSC could serve as a “cause of causes”, reducing workplace stressors and enhancing employee well-being.

Given our study results, the partial mediating role of relational stressors highlights the significance of interpersonal dynamics in healthcare settings, particularly those related to patient interactions, emphasizing the need for fostering positive relationships and communication channels within organizations to mitigate emotional exhaustion. Thus, our study contributes to the understanding of how relational stressors influences nurses' well-being and underscores the importance of intervention strategies aimed at reducing exhaustion, with a focus on enhancing the psychological safety climate and addressing relational stressors. This dual approach not only benefits the individual nurses but also improves overall organizational health and patient care quality.

### 4.1. Limitations

Several limitations should be acknowledged in our study. First, its cross-sectional design precludes causal inferences, necessitating longitudinal research to establish temporal relationships. Second, reliance on self-report measures introduces the potential for bias, suggesting the need for validation using objective measures or observational methods. Furthermore, our sample consisted solely of nurses from Italian hospitals, who were self-selected. This self-selection may not be representative of the entire Italian nursing population, limiting the generalizability of findings to other contexts.

### 4.2. Theoretical implications

Our study contributes theoretically by highlighting the role of psychological safety climate in mitigating relational stressors and emotional exhaustion among nurses. By identifying the perception of relational stressors as a mediator, our findings underscore the importance of interpersonal dynamics in influencing nurses' well-being. Additionally, our study adds to the literature on burnout in healthcare by elucidating the mechanisms through which organizational factors impact nurses' emotional exhaustion.

### 4.3. Practical implications

On a practical side, our findings emphasize the importance of promoting a positive psychological safety climate within healthcare organizations. This involves implementing policies and practices that prioritize nurses' well-being, such as communication training for managers and leadership interventions stimulating positive changes within wards [43]. On the other hand, also interventions involving nurses and targeting the perception of relational stressors, such as debriefing sessions and peer support programs [2], can further support nurses' mental health and resilience, thus working on the reduction of their risk of developing exhaustion. By prioritizing the creation of a supportive and safety-oriented organizational culture, healthcare organizations can empower nurses to effectively manage relational stressors and fulfil their professional roles with resilience. In doing so, they not only safeguard the well-being of their nursing staff but they also contribute to enhance the conditions that can ensure the overall quality and safety of patient care delivery.

### 4.4. Future research

Future research should address the current limitations of this study and explore additional job characteristics beyond relational stressors. Studies should examine PSC more profoundly, considering it both as an organizational level construct [21],[42] and from the perspective of perceived versus enacted PSC. Addressing PSC as an organizational-level construct in future research could provide a more comprehensive understanding of its impact. Aggregating PSC scores at the group or organizational level would allow for examining how shared perceptions of a supportive safety climate influence relational stressors and emotional exhaustion collectively. This approach could reveal more about the systemic factors that contribute to nurse well-being and highlight organizational interventions that could mitigate stress and burnout more effectively. About the perspective of enacted PSC, this could involve evaluating how organizational policies and leadership behaviors align with nurses' perceptions of PSC.

Moreover, future studies should investigate workload, autonomy, and job control, to understand their interaction with PSC and their impact on nurse well-being and patient care outcomes. In addition, given that our study was conducted in Italy, it would be valuable to replicate this research in different cultural contexts to examine whether the findings hold across various healthcare systems and cultural settings. Finally, future research should use randomized sampling methods to obtain a more representative sample of nurses, and employ longitudinal designs to establish causality between PSC, relational stressors, and emotional exhaustion. This last aspect would provide a clearer understanding of the temporal dynamics between these variables.

## 5. Conclusions

Our study underscores the critical role of psychological safety climate in mitigating exhaustion risk among nurses. In light of the study results, nurses working in a positive PSC perceive fewer social, relational stressors, which directly impacts their levels of emotional exhaustion. This underscores the importance of investing in safety-oriented organizational policies and practices. The mediation analysis further revealed that relational stressors partially mediated the relationship between PSC and emotional exhaustion, highlighting the role of the perception related to the risks involving interpersonal dynamics in influencing nurse burnout.

The implications of these findings suggest that a supportive PSC not only helps in reducing immediate perceived stressors but also fosters a work environment that sustains long-term nurse well-being. These benefits highlight the value of investing in comprehensive safety management systems in hospital settings.

## Use of AI tools declaration

The authors state that they used ChatGPT as an artificial intelligence (AI) tool for this study for the sole purpose of improving the written English of this article.
